# Laparoscopic surgery and coronavirus disease: What do we know now?

**DOI:** 10.6061/clinics/2020/e2083

**Published:** 2020-07-27

**Authors:** Sergio Conti Ribeiro, Ana Luisa F. Lauletta, Beatriz Couto Franco, Renata L Araujo Bezerra, Diana G B Salles Vanni, Edmund C. Baracat

**Affiliations:** Disciplina de Ginecologia, Departamento de Obstetricia e Ginecologia, Hospital das Clinicas HCFMUSP, Faculdade de Medicina, Universidade de Sao Paulo, Sao Paulo, SP, BR

**Keywords:** Coronavirus, Laparoscopy, Surgery

## Abstract

Questions regarding the transmissibility of the novel coronavirus disease (COVID-19) remain unanswered. It is known that the transmission of the severe acute respiratory syndrome coronavirus (SARS-CoV) occurs predominantly through droplets and contact. However, aerosols can be generated in some situations, such as orotracheal intubation, ventilation, and the use of electric or ultrasonic scalpels, and can therefore potentially contaminate the care team if adequate protection is not used. It is therefore necessary to assess issues of transmissibility of COVID-19 during surgery in infected patients. This review gathers the recent research pertaining to this topic. A search of the literature was performed using the PubMed and UpToDate databases with the search terms “surgery” and “covid-2019,” in addition to other MeSH variants of these terms. We do not have consistent evidence on the exposure of healthcare professionals assisting patients with COVID-19 undergoing laparoscopy or the impact of such exposure. In view of the evidence obtained and drawing parallels with other infectious and contagious diseases, medical personnel must wear complete protective attire for proper protection against the generated aerosol. Further studies are required to assess the impact of such surgeries on healthcare professionals conducing or assisting with these procedures.

The novel coronavirus disease (COVID-19) pandemic has taken the global health infrastructure by storm, and many healthcare systems are ill-prepared to cope with the rapid spread of the disease. In addition, instant access to information on the web and social media globally has created an unprecedented demand by the general population for transparency regarding infection rates, modes of transmission, and safety procedures upon hospital admission. In this context, health services are under high pressure to develop procedures to reduce the rising infection rates among healthcare professionals.

The new coronavirus, severe acute respiratory syndrome coronavirus 2 (SARS-CoV-2) has an average incubation period of about 5.5 days, with an interval of 0 to 14 days (1). Recent evidence confirms that about 80% of patients have asymptomatic or mild illnesses and that the average age of patients is less than 60 years (2). The basic reproductive value (R0) of COVID‐19 at the early stage was calculated to be between 2 and 3.5, indicating that one patient could transmit the disease to two or three other people, which was higher than the R0 calculated for SARS and MERS (3). SARS-CoV-2 RNA can be detected by reverse-transcription polymerase chain reaction (RT-PCR) although the duration of viral shedding is variable, depending on the severity of illness. In one study, viral RNA tests were negative after 10 days in patients with mild illness, whereas those with more severe illness had positive tests for longer. However, it is possible that even if viral RNA levels are sufficient for a positive RT-PCR result, infectivity may be unlikely because of the absence of infectious viral particles (4). Hence, the current model of hospital care proved inadequate to contain the nosocomial spread of the COVID-19 pandemic, which became evident after the complete closure of hospitals in Italy because of the high infection rates among doctors and nurses. By March 22, 2020, 4824 healthcare workers in Italy had been infected (9% of total cases), and 24 doctors had died. In China, on the same date, 3300 healthcare workers had been infected, and 23 doctors had died (5).

In an effort to prevent a massive health system overload, the Centers for Disease Control and Prevention, followed by the American College of Surgeons, recommended, at the beginning of the pandemic, the suspension of elective surgeries, as well as general precautionary measures for operating theater staff. Surgical interventions were subsequently restricted to patients with rapidly progressing malignancies or with active symptoms that required urgent care (2,6,7). Several months later, as further postponement of elective procedures is becoming a major public health concern, the question of how to adequately protect both staff and patients when operating on a potentially contaminated individual remains.

Initially, our research was based on a survey of the PubMed and UpToDate databases using the search terms “surgery” and “covid-2019,” in addition to MeSH variants of these terms. Unfortunately, only few articles were obtained. Because of the lack of controlled and retrospective studies on this recent pandemic, we chose to integrate our research results with those of similar studies, recommendations from surgical societies, and studies from countries in which the effects of the pandemic have already covered time and sample space. For this study we seek the main references reported in the most recent and comprehensive studies in the area. The review was conducted in full accordance with the recommendations of our Institutional Review Board.

What we know about the virology of SARS-CoV-2 and the disease, COVID-19, is that the RNA virus has a size range of 0.06-0.14 microns and is sensitive to ultraviolet radiation and heat (8,9,10). The virus is inactivated by incubation at 56°C for 30 minutes and by application of lipid solvents, such as ether, 75% ethanol, chlorine-containing disinfectant, peracetic acid, and chloroform; however, chlorhexidine is ineffective (10).

In previous studies on the transmissibility of infectious agents during laparoscopic surgeries, activated corynebacterium, human papillomavirus, HIV, and hepatitis B virus were detected in surgical smoke (11,12). Therefore, the risk of COVID-19 transmission also needs to be considered.

In laparoscopic surgery, special attention should be paid to several steps of the procedure, including orotracheal intubation, ventilation, establishment and maintenance of the pneumoperitoneum, use of electrical and ultrasonic scalpels, smoke evacuation, removal of specimens, pneumoperitoneum reversal, removal of trocars, and incisions closure.

The establishment and maintenance of an artificial pneumoperitoneum is a fundamental step in laparoscopic surgery. It is also very common to use ultrasonic scalpels or electrical equipment. These devices and the pneumoperitoneum produce a large amount of smoke, with the ultrasonic scalpels especially producing low-temperature aerosols (11,13). The viral components are not effectively deactivated by these sources of energy (11). Several energy sources (monopolar, bipolar, or ultrasonic) are also used in laparotomic surgery and produce smoke that can possibly infect the staff (14).

To effectively control surgical smoke during laparoscopic procedures, a combination of adequate air changes in the operating room and local exhaust ventilation (laparoscopic smoke filtering devices that remove surgical smoke from the peritoneal cavity) must be used to protect healthcare professionals and patients from exposure (12).

To control artificial pneumoperitoneum, the intraoperative pressure of the pneumoperitoneum and ventilation with CO_2_ should be kept at the lowest possible levels, without compromising the exposure of the surgical field. The reduction in the positioning time in Trendelenburg minimizes the effect of pneumoperitoneum on pulmonary function and circulation, thus increasing susceptibility to pathogens. Studies on laparoscopic surgery in patients with HIV, Hepatitis B and HPV found that the energy settings of the electrocautery should be as low as possible and that lengthy dissection at the same site would increase surgical smoke (11). Such smoke, produced with or without heat, contains aerosols with both viable and non-viable cellular material, which subsequently presents a risk of infection and causes irritation in the lungs, leading to acute and chronic inflammatory changes, thus also increasing susceptibility (12).

After using electrical or ultrasonic equipment for 10 minutes, the concentration of particles in the smoke from laparoscopic surgery is significantly higher than that in traditional open surgery. Because of the low mobility of gases in the pneumoperitoneum, the aerosols formed during the operation tend to be concentrated in the abdominal cavity, thereby increasing the risk when using the laparoscopic technique in comparison to that when using traditional open surgery. The sudden release of trocar valves, clamp changes, or even small abdominal wall incisions can potentially expose the OR team to the pneumoperitoneum aerosol. This outbreak, therefore, represents a major challenge to the clinical work of surgeons who practice minimally invasive surgery (11).

Electrocautery and the use of laser systems involve the same mechanism of generating surgical smoke. During the procedure (cutting, coagulating, vaporizing, or removing tissues), heating of the target cells to the point of injury causes membrane rupture and dispersion of the fine claws in the air or pneumoperitoneum, depending the ultrasonic scalpels used; this heating process is called “low-temperature vaporization.” The smoke generated by this process has a greater chance of carrying viable and infectious loads than do high-temperature aerosols (13).

The average size of the particles generated varies widely, depending on the energy method used. Electrocautery creates particles with the smallest average aerodynamic size (0.1 mm), laser tissue ablation creates larger particles (0.3 mm), whereas the largest particles are generated by ultrasonic scalpels (0.35 to 6 mm). These particles travel greater distances from the point of production (up to 100 cm). Particles of 0.5 to 5.0 mm can penetrate the lung, inducing acute and chronic inflammatory changes, including alveolar congestion, interstitial pneumonia, bronchiolitis, and emphysematous changes in the respiratory tract. Surgical smoke has also been shown to be cytotoxic, genotoxic, and mutagenic. The mutagenic effect created by the temperature used for the destruction of 1 g of tissue in the laser and electrocautery methods is equivalent to that of three and six cigarettes, respectively (13).

Before making the auxiliary incision for removal of specimens, the gas in the abdominal cavity should be exhausted as much as possible to prevent massive injection of residual gas. Postoperative specimens are potentially infective and should be treated carefully when handed over to the pathology department for treatment. Special attention should be paid to evacuating residual CO_2_ from the container and the abdominal cavity before removing the trocars (15).

Surgery is ideally performed in a designated negative pressure or infection surgery room with minimal participants and a prominent sign posted on the door. The operating room should be equipped with a thermometer to monitor the temperature of each medical staff before surgery (7). The risk of exposure is cumulative and is greater for those closer to the point of smoke production (13). Intraoperatively, filters such as high-efficiency particulate air filters are used to remove smoke and particulate matter, including viruses. These have a 99.97% efficiency rate for removing particles as small as 0.3 microns in diameter (12). Another filter that can be used is the ultra-low particulate air filter, which can remove 99.999% of airborne particles greater than or equal to 0.05 microns in size (9). The N95 respirator mask filters out 95% of the particles that are 0.3 microns or larger.

SARS-CoV-2 viral particles are found within the cells lining the gastrointestinal and respiratory tracts, suggesting that the virus has multiple modes of transmission (7,9). Thus, it is recommended that care be taken universally when performing endotracheal intubation and ventilation and when managing body fluids.

For procedures that cannot be postponed, considering the possibility of viral contamination during laparoscopy is highly recommended (2,7). This risk must be considered individually against the benefit of laparoscopy for a patient's health and recovery. Although this has not been confirmed for coronavirus, on the basis of studies on other viral infections, it is safe to presume that this virus can be released with carbon dioxide during laparoscopy.

Routine preoperative PCR testing for COVID-19 in all surgical patients is important to detect possible asymptomatic transmitters. In the case of oncologic and other important elective surgeries that cannot wait for the end of the pandemic, waiting at least for a negative viral PCR result before performing the procedure can provide additional safety, as the surgery will generate relative immunosuppression and may render the patient more susceptible to infection.

Currently, we do not have consistent evidence on the exposure of healthcare professionals who assist patients with COVID-19 undergoing laparoscopy and the impact of such exposure. In view of the evidence obtained and drawing parallels with other infectious and contagious diseases, medical personnel must wear complete protective attire for proper protection against the generated aerosol. Further studies are required to assess the impact of such surgeries on healthcare professionals conducing or assisting with these procedures. As we stated before, the use of an electrosurgical unit can also create aerosol suspension in laparotomic procedures and potentially spread COVID-19 all over the operating room. In view of the benefits of laparoscopy over laparotomy in terms of shorter hospital stay, greater recovery speed and less inflammatory stress, we recommend that laparoscopic surgery performed with the appropriate protective equipment and a pneumoperitoneal outlet filter is still more beneficial than laparotomy surgery.

According to the evidence acquired so far, and based on SOBRACIL recommendations for protecting surgical teams (14), we propose the following measures: performance of COVID-19 testing in patients and the surgical team before any procedure, avoidance of pneumoperitoneum leakage using a specific filter, use of appropriate two-way protective apparel, removal of the protective apparel using the appropriate technique, hand washing and showering before leaving the hospital, if possible.

## AUTHOR CONTRIBUTIONS

Ribeiro SC was responsible for the study conception and design and critical revision of the manuscript. Lauletta ALF was responsible for the study conception and design, manuscript drafting, and critical revision. Franco BC and Bezerra RLA were responsible for the study conception and design and for manuscript drafting. Vanni DGBS and Baracat EC were responsible for critical revision of the manuscript.
